# A Network Equivalent-Based Algorithm for Adaptive Parameter Tuning in 802.15.4 WSNs

**DOI:** 10.3390/s18072031

**Published:** 2018-06-25

**Authors:** Yipeng Wang, Wei Yang, Ruisong Han, Kaiming You

**Affiliations:** 1 School of Electronic and Information Engineering, Beijing Jiaotong University, Beijing 100044, China; 14111029@bjtu.edu.cn (Y.W.); youkaiming@bjtu.edu.cn (K.Y.); 2 Telecommunications Software & Systems Group (TSSG), Waterford Institute of Technology, X91 P20H Waterford, Ireland; rhan@tssg.org

**Keywords:** wireless sensor networks, IEEE 802.15.4, CSMA/CA, reliability

## Abstract

Previous studies have shown that in many wireless sensor network applications the IEEE 802.15.4 carrier sense multiple access with collision avoidance (CSMA/CA) mechanism with default parameters cannot guarantee the constraints of reliability, time efficiency, or energy efficiency. Although many adaptive parameter tuning algorithms have been proposed, many of them cannot correctly identify the changes of the network condition and are unable to effectively perform the parameter tuning operation. Considering the randomness that CSMA/CA brings about, for most of the proposed algorithms, it is a challenge to distinguish significant violations that were caused by actual changes of the network from the general fluctuations that were due to CSMA/CA. In this paper, we propose a lightweight algorithm called the network equivalent adaptive parameter tuning (NEAPT) algorithm. It is fully distributed and can work without any predefined information or acknowledgement. NEAPT not only takes reliability as an evaluation of a network condition, but it proposes a synthetic value, called the *equivalent node number*, and takes it as another reference for a network condition. Simulation results show that by taking both reliability and the *equivalent node number* into consideration, NEAPT can effectively identify the network changes and provide adequate and steady performances for wireless sensor networks (WSNs) in both stationary and dynamic conditions.

## 1. Introduction

Wireless sensor networks (WSNs), as one kind of personal area networks (PANs), have penetrated various applications, from habitat monitoring to industrial control, for their advantages of low deployment costs, easy installation, maintenance, and reconfiguration, as well as inherent intelligent-processing capability over traditional wired devices [1]. The IEEE 802.15.4 standard, which covers the physical and medium access control (MAC) layers’ specifications targeting the PANs, has also been thoroughly investigated for more than a decade. In this paper, we focus on the effect of IEEE 802.15.4 MAC layers’ channel access protocol on the whole network’s performance during the contention access period (CAP). For general WSNs applications, reliability, time efficiency, and energy efficiency are always specifically and strictly demanded. In addition, for some specific applications, other issues like robustness and security are also within their evaluation scopes, as listed by Salam et al. [2].

According to the IEEE 802.15.4 standard, general WSNs always adopt the slotted carrier sense multiple access with collision avoidance (CSMA/CA) algorithm as their channel access protocol during CAP. However, it has been shown by the authors of Anasasti et al. [3] that a slotted CSMA/CA algorithm cannot guarantee both energy efficiency and reliability constraints simultaneously with its parameter values held at the default. To avoid the current standards being modified, and in order to accommodate various applications, using adaptive parameter tuning algorithms is necessary.

According to the IEEE 802.15.4 standard, general WSNs always adopt the slotted carrier sense multiple access with collision avoidance (CSMA/CA) algorithm as their channel access protocol during CAP. However, it has been shown by Anasasti et al. [3] that a slotted CSMA/CA algorithm cannot guarantee both energy efficiency and reliability constraints simultaneously with its parameter values held at the default. To avoid the current standards being modified and to accommodate various applications, many proposals have been proposed. In the work of [4], Khanafer et al. provides a comprehensive study of the existing proposals for the MAC layer of IEEE 802.15.4 WSNs in beacon enable mode. And they categorize the main proposed solutions into eight different categories, namely priority-based, QoS-based, hidden node resolution-based, IEEE 802.11-based, duty cycle-based, backoff-based, parameter tuning-based, and cross layer-based.

Priority-based solutions consider the different urgency of traffics and prioritize node access to the medium accordingly [5,6,7]. QoS-based solutions aim at achieving a better utilization of the bandwidth. It is achieved by providing enhanced GTS mechanisms with better GTS allocation efficiency [8,9]. Hidden node resolution-based solutions consider the existence of hidden nodes and aim to reduce the number of collisions caused by hidden nodes [10,11]. IEEE 802.11-based approaches try to migrate some solutions that have proven efficient in IEEE 802.11 to 802.15.4 WSNs [12]. However, a lack of considering energy efficiency makes them not suitable for most of WSNs. Duty-cycle based approaches can provide better energy efficiency without compromising other important network performances. It is achieved by adjusting the duty-cycle of the sensor nodes to traffic conditions adaptively. However, it is not suitable for large scale WSNs with simultaneous transmission [13,14,15]. Backoff-based approaches try to improve the efficiency of medium access with adaptive and dynamic backoff algorithms [16,17]. Parameter tuning-based and cross layer-based solutions all rely on the MAC layer parameters configuration without modifying the current standard. The difference between the two categories is that the parameter tuning-based solutions perform parameter configurations according to information of its own layer (i.e., MAC layer) [18,19,20,21], while in cross layer-based solutions [22,23,24,25,26], parameter tunings are also based on other layers (e.g., physical layer or network layer). The cross layer-based solutions can be further classified as model-based [20,21,22] and measurements-based [23,24,25,26], according to the methodology for parameter tuning. Model-based strategies rely on an analytical model of the WSN to derive the optimal parameter setting under the current operating conditions. Measurement-based approaches do not require any network model, instead they rely on measurements acquired by sensor nodes. In this paper, the proposed algorithm, that is, the network equivalent adaptive parameter tuning (NEAPT) algorithm, belongs to this class.

Although many cross layer-based parameter tuning solutions have been proposed, there still exists several problems that should be discussed and specified regarding the evaluation of the network condition and the strategy of parameter tuning. According to Park et al. in [20], previous algorithms have paid more attention to the energy efficiency of sensor nodes, while explicit consideration of the application requirements like reliability and time efficiency have been relatively less mentioned. In [20], Park et al. proposed a Markov chain model-based adaptive algorithm for minimizing power consumption while satisfying the reliability and time efficiency requirements. However, the algorithm is impractical due to the necessity of a predefined network size and traffic pattern. An experience-based heuristic adaptive algorithm, ADAPT, was proposed by Francesco et al. [23] to address this. It is a classic measurements-based cross-layer parameter tuning solution and inspires many schemes [24,25,26], including NEAPT. Based on ADAPT, another experience-based heuristic adaptive algorithm, JIT-LEAP, was also proposed by authors of [24,25] for more stable and accurate parameter tuning performance. Although algorithms like those from the authors of [23,24,25] have proved to be accommodated with real-life scenarios and are able to provide accurate parameter settings, they have poor performance in terms of time efficiency and energy efficiency. Their reliance on the acknowledgement (ACK) mechanism brings extra overhead, waiting time, and energy consumption. In [26], the authors proposed a blind adaptive algorithm, called BADAPT, to be free from predefined parameters and the ACK mechanism. With BADAPT, each sensor node only needs to utilize locally measured information for the network condition’s evaluation and corresponding parameter tuning. Both ADAPT and BADAPT adopt two thresholds and the exponential moving average method to avoid tardy or excessive adaptation. According to their results, they are not always capable of distinguishing significant violations that were caused by actual changes of network condition from general violations, because of the randomness of the channel access mechanism. In stationary conditions, their senses of parameter tuning are easily affected by the randomness of CSMA/CA. In the dynamic conditions, the proposed ADAPT algorithm cannot sustain an acceptable performance under the sudden changes of the network condition, according to the simulation result of Francesco et al. [23], and neither does BADAPT in Zhang et al. [26].

This paper proposes a heuristic algorithm, called network equivalent adaptive parameter tuning (NEAPT), for better coping with both stationary conditions and dynamic conditions, without modifying the current standard or introducing an extra cost. Contrary to directly comparing the estimated reliability with the required reliability, this paper provides a different angle on network condition estimation. First, with the local measured information and current values of the key parameters, each sensor node can perform a conservative evaluation of the surrounding network’s condition. The result of the evaluation is presented as a synthetic value, called the equivalent node number. Then, considering the measured reliability and required reliability, as well as the equivalent node number, the phase of parameter tuning is considered regarding whether it will be active or not. The proposed NEAPT is fully distributed and can work without any predefined information and acknowledgement, as the evaluation phase and parameter tuning phase are individually performed by each sensor node. As will be shown, NEAPT is able to detect the actual changes in the network condition and distinguish them from the general violations in both stationary conditions and dynamic conditions.

The remainder of this paper is organized as follows: Section 2 describes the IEEE 802.15.4 slotted CSMA/CA mechanism. A simple analysis of each node’s reliability is given in Section 3. The proposition of *equivalent node number* and the specific procedure of NEAPT algorithm are presented in Section 4. Section 5 presents the simulation results. Concluding remarks are provided in Section 6.

## 2. IEEE 802.15.4 Slotted CSMA/CA

Sensor devices can operate in either of the two modes according to the IEEE 802.15.4 standard, (i.e., non-beacon-enabled mode and beacon-enabled mode) [27]. This paper focuses on the latter. In the beacon-enabled mode, time is divided into discrete time slots called the backoff period (BP) with a fixed duration of 320 μs. The PAN coordinator periodically sends beacon frames for network synchronization. The time interval between two consecutive beacon frames is called the beacon interval (BI), which is decided by the value of the beacon order (BO), where 0 ≤ BO ≤ 14. The period of the BI is composed of an active period and an inactive period. The active period is structured as a superframe for data packet transmission. The superframe duration (SD) is determined by the value of the superframe order (SO), where 0 ≤ SO ≤ BO ≤ 14. Within one superframe, the first time slot is allocated for the beacon frame and the rest of the time slots are divided into contention access period (CAP) and contention free period (CFP).

The beacon period, CFP, and inactive period are beyond current consideration, as the slotted CSMA/CA mechanism is defined for channel access during CAP. According to slotted CSMA/CA, three parameters are initialized when the data packets need to be transmitted (i.e., the number of backoff stages (NB), the contention window (CW), and the backoff exponent (BE). A sensor node backs off for several time slots before each data packet’s transmission, called *aUnitBackoffPeriod*. The number of *aUnitBackoffPeriod* is randomly selected within (0, 2^BE^ − 1). The value of BE starts from *macMinBE*. After that, the sensor node performs CW_0_ times of the clear channel assessments (CCA), consecutively. If either CCA reveals that the channel is busy, then NB and BE are both increased by 1 and the CW is reset to CW_0_. The current packet transmission is identified as failed if the value of NB reaches its maximum value *macMaxCSMABackoffs*. If the value of BE reaches the maximum *macMaxBE*, then the sensor node will keep its value until a successful/failed packet transmission occurs or packet retransmission commences. Table 1 shows the default values of the slotted CSMA/CA parameters according to IEEE 802.15.4.

## 3. Reliability Analysis

The WSNs’ network traffic can be assumed to be a Poisson process [28], as some research has previously proved. In this paper, we will maintain this assumption and further exploit the properties of the Poisson process for a more accurate evaluation. In the following analysis, we consider a single-hop WSN with star topology, which is always directly applied to group-based monitor applications or exists inside clusters in larger scale WSNs. In the network we considered, one sink node acts as a PAN coordinator and multiple sensor nodes are deployed around the PAN coordinator within each other’s carrier sensing range. Therefore, there are no hidden terminals in the network. To access channels with slotted CSMA/CA, the sensor nodes are assumed to work in a beacon enabled mode, and to transmit the data packet without a handshaking mechanism. The CW_0_ times of CCA are assumed to be 1 successive CCA. So that all discarded packets are due to either a channel access failure or data packet collision, we assume perfect channel conditions and infinite queue length.

For each sensor node, we assume the delivery ratio as the index of reliability, which is defined as the ratio between the number of data packets successfully transmitted by a sensor node and the total number of packets generated by the sensor node. As mentioned above, to successfully transmit a data packet in slotted CSMA/CA without ACKs, sensor nodes should achieve successful CCA, and avoid collision at the same time. Hence, the probability of successful data packet transmission, $p_{}^{S}$, can be derived as follows:

(1) $$p^{S}=p_{CCA}^{S}\cdot p_{CA}^{S},$$

where $p_{CCA}^{S}$ is the probability of successful CCA and $p_{CA}^{S}$ is the probability of no collision occurring during transmission.

Every time a sensor node has a data packet to be transmitted, its MAC layer begins to perform the CCA attempt. A new CCA process is performed right after the previous CCA process fails, until the current CCA is a success or *macMaxCSMABackoffs* is reached. The expected aggregate arrival rate of the CCA attempts $\lambda_{CCA}$ can be represented in terms of λ, as follows:

(2) $$\lambda_{CCA}=\lambda \cdot \sum_{k=0}^{N_{B}^{\left(\max\right)}}{\left(1-p_{CCA}^{S}\right)}^{k},$$

where λ is the aggregate arrival rate of network and $N_{B}^{\left(\max\right)}$ is the value of *macMaxCSMABackoffs*. 

The CCA attempt rate $\lambda_{CCA}$ also follows the Poisson distribution with fixed $N_{B}^{\left(\max\right)}$ and $p_{CCA}^{S}$, since the packet arrival rate is assumed to follow the Poisson distribution.

**Figure 1:**
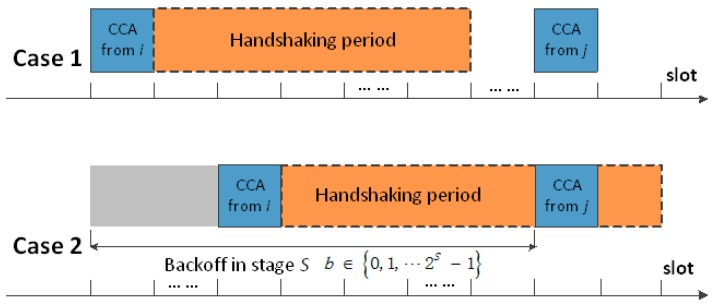
Two kinds of successful clear channel assessments (CCA) processes.

According to the content of the slotted CSMA/CA algorithm, before transmission, the sensor node should perform the CCA process to check whether the channel is idle or not. If the channel is busy, it will back off for a random number of *aUnitBackoffPeriod* and before checking again. Nodes can avoid the failure of CCA to some extent, with the assistance of backoff. A larger NB offers more chances for CCA. A larger BE offers nodes more backoff delay options, which enlarges the disparity in the backoff duration among nodes to decrease the probability of CCA failure. The IEEE 802.15.4 slotted CSMA/CA algorithm bounds the value of the backoff exponent between *macMinBE* $BE_{\min}$ to *macMaxBE*. $BE_{\max}$. It should be noted that the maximum value allowed for $BE_{\min}$ by the IEEE 802.15.4 standard is equal to 8 [27]. Hence, the probability of successfully passing CCA $p_{CCA}^{S}$ is affected by $N_{B}^{\left(\max\right)}$ and $BE_{\min}$. Assuming that all data packets are of the same packet size, they correspond to occupying the same channel time of $n_{PKT}$ slots. For each sensor node, successful CCAs on its targeted channel mainly happen in two cases, as shown in Figure 1.

According to Case 1, if the time interval between the current CCA and the previous CCA on the targeted channel is larger than $n_{PKT}$ slots, the current CCA will be successful, because the time interval is enough for the previous transmission. According to the theory of Poisson distribution [29], we can find the time interval between two consecutive CCAs by following an exponential distribution. Therefore, the probability of Case 1 can be expressed as follows:

(3) $$p_{CCA}^{CI}=p[T_{n}\geq n_{PKT}\cdot T_{slot}]=e^{-\lambda_{CCA}\cdot T_{PKT}},$$

where $T_{slot}$ is the time duration of *aBaseSlotDuration*.

As shown by Case 2 in Figure 1, if the time interval between a sensor node’s current CCA and its targeted channel’s previous CCA is not larger than $n_{PKT}$ slots, then the sensor node can take its own previous backoff period as a reference to do a primary evaluation. Supposing that all nodes are staying at the same backoff stage S, one node’s CCA attempt can be successful only if no CCA attempts succeed within the previous $n_{PKT}$ slots. Hence, the probability of Case 2 $p_{CCA}^{BE}$ can be obtained from the following:

(4) $$p_{CCA}^{BE}=1-[1-{\left(1-\frac{1}{2^{BE}-1}\right)}^{N_{C}-1}]\cdot p_{CCA}^{BE}\cdot \frac{n_{PKT}\cdot T_{slot}}{T_{bkunit}},$$

where $T_{bkunit}$ is the time duration of *aUnitBackoffPeriod* and $N^{C}$ is the number of sensor nodes that are also targeting to the channel. Hence, the probability of achieving successful CCA can be expressed as follows:

(5) $$p_{CCA}^{S}=1-\prod_{i=0}^{N_{B}^{\left(\max\right)}}\left(1-(p_{CCA}^{CI}+(1-p_{CCA}^{CI})\cdot p_{CCA}^{BE}(i))\right).$$

Collisions can occur because of either the hidden node problem or when more than one node performs data packet transmission simultaneously. Since the hidden node problem is excluded by assumption, collisions can only result from simultaneous transmissions. According to the slotted CSMA/CA algorithm, a node will transmit its data packet immediately after the channel is found to be idle in CCAs. Hence, simultaneous transmission happens right after two or more devices pass CCAs synchronously. The probability of no collision happening can be approximated as follows:

(6) $$p_{CA}^{S}=e^{-(p_{CCA}^{S}\cdot \lambda_{CCA})T_{bkUNIT}}.$$

Therefore, by substituting Equations (5) and (6) into Equation (1), the probability of successful data packet transmission $p^{S}$ can be obtained, as follows:

(7) $$p^{S}=\left(1-\prod_{i=0}^{N_{B}^{\left(\max\right)}}\left(1-(p_{CCA}^{CI}+(1-p_{CCA}^{CI})\cdot p_{CCA}^{BE}(i))\right)\right)\cdot e^{-(p_{CCA}^{S}\cdot \lambda_{CCA})T_{bkUNIT}}.$$

A brief presentation of Equation (7) can be presented as follows:

(8) $$p^{S}=\varphi (N_{C},\lambda ,n_{PKT},N_{B}^{\left(\max\right)},BE_{\min},BE_{\max}).$$

## 4. Network Equivalent Adaptive Tuning Algorithm

To our best knowledge, all previously known parameter tuning algorithms take the reliability index as their basis of parameter tuning. However, we think that only taking either the historical reliability or the local estimated reliability as the evaluation criterion of the network condition is actually acting based on the symptom, not the problem. In each IEEE 802.15.4 based WSN, the changes of the sensor node’s packet delivery ratio not only come from the actual changes of the network condition, but may also be caused by the randomness of the channel accessing mechanism. Considering that one WSN stays in a stationary scenario, that is, all senor nodes are fixed, no sensor nodes are added/removed, and no traffic pattern changes happen. The value of each node’s packet delivery ratio still cannot be fixed for the randomness of the medium access mechanism. A wise parameter tuning algorithm should be able to distinguish significant violations that are caused by actual changes of network condition, from general fluctuations that are due to the randomness of channel access mechanism, as well as be able to cope with the changes of reliability that come with a divide-and-conquer strategy.

**Figure 2:**
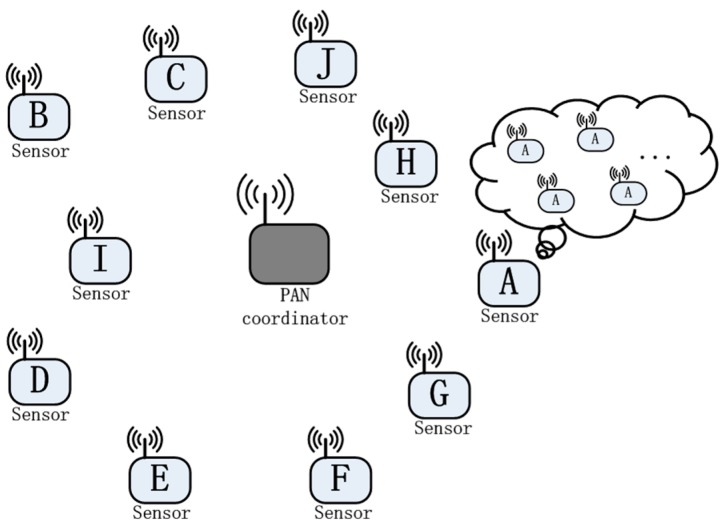
The sensor node’s equivalent process of surrounding network status.

Based on the reliability analysis in the previous section, each sensor node can perform a conservative evaluation of the surrounding network’s condition with the local measured packet delivery ratio and current values of the key parameters. At first, each sensor node assumes that its surrounding is composed of one kind of sensor nodes with the same traffic patterns and parameter settings as itself, as shown in Figure 2. We call these sensor nodes equivalent nodes.

Concerning an inverse function of Equation (8) with $N_{i}^{C}$ as the output, as follows:

(9) $$N_{C}=\varphi^{-1}(p^{S},\lambda ,n_{PKT},N_{B}^{\left(\max\right)},BE_{\min},BE_{\max}),$$

For each sensor node, at the end of the *i*th BI, by substituting locally measured packet delivery ratio $p_{i}^{S}$, the arrival rate of own data packet λ, transmission time of each data packet $n_{PKT}$ and current values of self-parameters into Equation (9), a corresponding $N_{i}^{C}$ can be obtained. The value of the obtained $N_{i}^{C}$ can be taken as the *equivalent node number* that the sensor node evaluated at the end of *i*th BI, marked as ${\tilde{N}}_{i}^{C}$. Similar to ADAPT [6] and BADAPT [8], we also define the required reliability as $p_{req}^{S}$. The value of $p_{req}^{S}$ is different when subjected to different WSNs applications, and should be claimed before the execution of the parameter tuning. Accordingly, with specified $p_{req}^{S}$, the value of *equivalent node number* ${\tilde{N}}_{req}^{C}$ can also be obtained by applying $p_{req}^{S}$, own data packet arrival rate λ, transmission time of each data packet $n_{PKT}$, and self-parameters into Equation (9). At the end of each BI, each sensor node decides whether and how to perform the parameter tuning process by comparing $p_{i}^{S}$ and ${\tilde{N}}_{i}^{C}$ with $p_{req}^{S}$ and ${\tilde{N}}_{req}^{C}$. The specific processes of NEAPT are presented in Algorithm 1.
**Algorithm 1** NEAPT.**1**     **if**
$p_{i}^{S}<p_{req}^{S}$ then**2**      **if**
$\left|{\tilde{N}}_{i}^{C}-{\tilde{N}}_{req}^{C}\right|>\delta_{N}$ then**3**       **if**
*macMinBE* < *macMinBE^max^*
**then****4**        *macMinBE* ++**5**       **else if**
*macMaxCSMABackoffs* < *macMaxCSMABackoffs^max^*
**then****6**        *macMaxCSMABackoffs* ++**7**       Update ${\tilde{N}}_{req}^{C}$
**8**      **else if**
$\left|{\tilde{N}}_{i}^{C}-{\tilde{N}}_{req}^{C}\right|\leq \delta_{N}$ then**9**        *macMinBE* = *macMinBE***10**        *macMaxCSMABackoffs* = *macMaxCSMABackoffs***11**     **else if**
$p_{i}^{S}\geq p_{req}^{S}$ then**12**      **if**
$\left|{\tilde{N}}_{i}^{C}-{\tilde{N}}_{req}^{C}\right|>\delta_{N}$ then**13**       **if**
*macMaxCSMABackoffs* > *macMaxCSMABackoffs^min^*
**then****14**        *macMaxCSMABackoffs* --**15**       **else if**
*macMinBE* > *macMinBE ^min^*
**then****16**        *macMinBE* --**17**       Update ${\tilde{N}}_{req}^{C}$
**18**      **if**
$\left|{\tilde{N}}_{i}^{C}-{\tilde{N}}_{req}^{C}\right|\leq \delta_{N}$ then**19**        *macMinBE* = *macMinBE***20**        *macMaxCSMABackoffs* = *macMaxCSMABackoffs*

At the end of every BI (take the *i*th BI as an example), each node compares its local measured packet delivery ratio $p_{i}^{S}$ with $p_{req}^{S}$. If $p_{i}^{S}$ is smaller than $p_{req}^{S}$, then the sensor node will calculate and compare ${\tilde{N}}_{i}^{C}$ with ${\tilde{N}}_{req}^{C}$. If the difference between ${\tilde{N}}_{i}^{C}$ and ${\tilde{N}}_{req}^{C}$ is greater than the threshold of the network change $\delta_{N}$, then the *macMinBE* is increased until *macMinBE^max^* is reached. Then, *macMaxCSMABackoffs* is increased until *macMaxCSMABackoffs^max^* is reached. After that, the value of ${\tilde{N}}_{req}^{C}$ is recalculated and updated. If the difference between ${\tilde{N}}_{i}^{C}$ and ${\tilde{N}}_{req}^{C}$ is within the threshold, the sensor node holds the current ${\tilde{N}}_{req}^{C}$ value and current parameter settings. If $p_{i}^{S}$ is not less than $p_{req}^{S}$ and the difference between ${\tilde{N}}_{i}^{C}$ and ${\tilde{N}}_{req}^{C}$ is greater than $\delta_{N}$, then *macMaxCSMABackoffs* is first decreased, until *macMaxCSMABackoffs^min^* is reached, and then *macMinBE* is decreased until *macMinBE^min^* is reached. Next, the value of ${\tilde{N}}_{req}^{C}$ is recalculated and updated. If the difference between ${\tilde{N}}_{i}^{C}$ and ${\tilde{N}}_{req}^{C}$ is within the threshold, then the sensor node holds the current ${\tilde{N}}_{req}^{C}$ value and current parameter settings. As shown from the above description, NEAPT is not as flexible as ADAPT or BADAPT with the addition of the *equivalent node number* threshold.

## 5. Simulations

For the simulation, we used the OMNET++ simulator [30] to evaluate the performance of NEAPT in both stationary and dynamic scenarios. According to the authors of [6,7,8], we made the simulation with a setup, as listed in Table 2. In all the experiments, we assumed that all sensor nodes were operating on top of the 2.4 GHz Industrial, Scientific, and Medical (ISM) radio band, with a maximum bit rate of 250 Kbps. All the sensor nodes’ default parameter settings, operation latency, and power consumptions were according to the IEEE 802.15.4 standard [27] and MICAz mote specification [31], to generally be as close to the actual as possible. Similar to the authors of [8], we also put the sensor nodes to sleep when they had no packets ready for transmission, and to idle when they were in backoff. We consider that sensor nodes were randomly placed 10 m away from the PAN coordinator. The transmission range was set to 15 m, while the carrier sensing range was set to 30 m, according to the authors of [8]. The BI was 125.8 s (i.e., BO = 13), and the active period was set to SD = 15.72 s (i.e., SO = 10). Each sensor node was assumed to generate data packets with different but fixed time intervals, which ranged from 1 s to 5 s. For one channel that is shared by several assumed sensor nodes, it is not hard to prove that the aggregated packet arrival rate of the shared channel follows the Poisson distribution. The proof, in detail is appended in Appendix A.

In addition to the packet delivery ratio, two other distinct observables were also included for the performance evaluation, namely: **(1) Average latency**, which is defined as the time duration from the instant that one packet was at the head of the MAC queue, to when the packet was successfully transmitted. This is the index of time efficiency. The latency mainly comes from the backoff operations on the MAC layer; and **(2) Average energy consumption**, which is defined as the average power of successfully transmitting one data packet. It is the index of energy efficiency. The energy consumption is mainly composed of the performing CCA, backoff, and data packet transmission.

Besides NEAPT, another three parameter tuning algorithms were taken into consideration for the following evaluation, namley: the default parameters set (DPS) (i.e., the default values specified by the IEEE 802.15.4 standard), ADAPT from the authors of [6], and BADAPT from the authors of [8], as the newest measurements-based cross-layer parameter tuning parameter tuning algorithm. Notice that in [6], the ACK mechanism was adopted in ADAPT for reliability estimation. Although employing ACKs can save some data packets from collision, it comes at the cost of additional latency and increased power consumption. In the following analysis, DPS is compared as the baseline algorithm and is presented as a blue curve; ADAPT is compared as the classic measurements-based cross-layer parameter tuning algorithm and presented as a blue curve with blue bubbles; BADAPT is compared as the newest measurements-based cross-layer parameter tuning algorithm and presented as a black curve with black bubbles. The proposed NEAPT algorithm is presented as a red curve with red asterisks.

### 5.1. Analysis in Stationary Conditions

**Figure 3:**
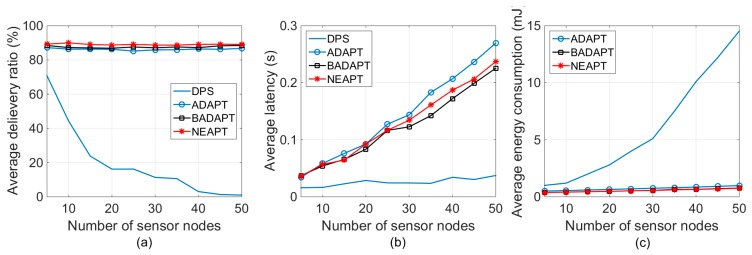
Delivery ratio, latency and energy consumption under stationary conditions. (**a**) Average delivery ratio. (**b**) Average latency. (**c**) Average energy consumption.

This section focuses on the stationary scenarios with a fixed number of active sensor nodes. In this part, different network scales were evaluated from 5 sensor nodes to 50 sensor nodes. From Figure 3a, we can observe that NEAPT was able to meet the reliability requirements under different network scales, while a network with DPS suffered a sharp deterioration in reliability, as the network scale increased. As is shown in Figure 3b, NEAPT had a larger latency than DPS, since it inevitably brings more backoff chances and a longer waiting time. Specifically, a larger network scale brings a larger latency with NEAPT. As a result of autonomous adaptive parameter tuning, it can be seen that NEAPT offers a nearly constant average delivery ratio, which also brings a significant reduction in energy consumption compared with the DPS, as shown in Figure 3c.

The average packet delivery ratio of NEAPT is always higher than ADAPT and BADAPT, according to the results presented in Figure 3a. Although the style of parameter tuning that NEAPT provides is conservative and inflexible, Figure 3b shows that NEAPT had almost the same performance as BADAPT, in terms of latency—especially in networks with no more than 25 nodes. The average latency of taking NEAPT was always lower than taking ADAPT. Moreover, considering energy consumption, adopting NEAPT could save 9.72% of the energy consumption than if adopting BADAPT. Compared with ADAPT, adopting NEAPT could save 16.53% of the energy consumption. This additional improvement is mainly benefited from not using ACKs.

**Figure 4:**
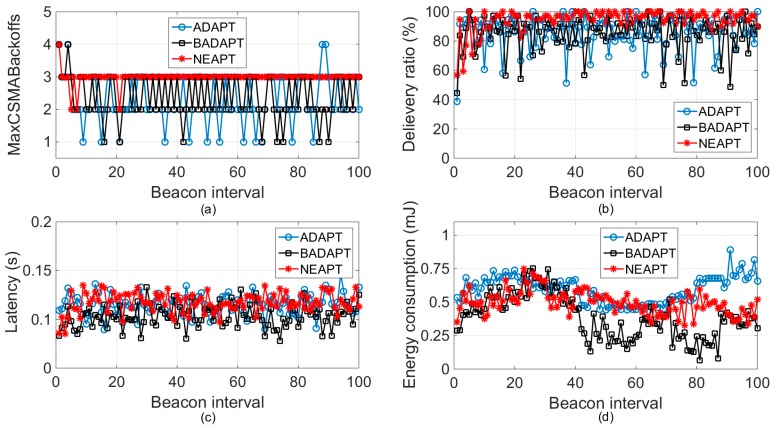
Parameter tuning results and corresponding delivery ratio, energy consumption, and latency with 25 sensor nodes. (**a**) *macMaxCSMABackoffs*. (**b**) Delivery ratio. (**c**) Latency. (**d**) Energy consumption.

An experiment with 25 active sensor nodes was performed to gain a more concrete understanding of NEAPT. This experiment was also in a stationary condition and its execution time was 100 BIs. We focused on 1 of 25 sensor nodes and recorded its observable performance as well as its value of *macMaxCSMABackoffs*. As shown in Figure 4a, the value of *macMaxCSMABackoffs* with NEAPT always equaled to 3. Whereas with ADAPT and BADAPT, the value of *macMaxCSMABackoffs* changed very frequently. More specifically, within the considered 100 BIs, the total number of fluctuations for NEAPT was 7. While for ADAPT and BADAPT, the total number of fluctuations were 70 and 81, respectively, which is 10 times and 11.57 times, respectively, that of NEAPT. However, Figure 4b indicates that NEAPT could provide higher and steadier reliability performances than the other two algorithms. By combining Figure 4a and Figure 4b, we can find that NEAPT was able to avoid more unnecessary tuning than ADAPT and BADAPT. This is due to the conservative evaluation of the local network condition analysis and network node equalization that NEAPT provides. Similarly, Figure 4c,d also shows that NEAPT had a more adequate and steadier performance, in terms of energy consumption and time efficiency, than the other two algorithms. The mean value and variation of transmission latency that ADAPT and BADAPT provided were (131.406 ms, 0.557 ms) and (116.552 ms, 0.483 ms), separately. By adopting NEAPT, the mean value and variation of transmission latency were 107.72 ms and 0.403 ms, which was lower than ADAPT by 22.04% and 38% (and for BADAPT, 8.20% and 19.85%). Considering the randomness of CSMA/CA and the time varying characteristics of the wireless channel, this conservative and inflexible parameter tuning algorithm can protect the sensor nodes from being disturbed by tiny fluctuations of the packet delivery ratio.

### 5.2. Analysis in Dynamic Conditions

In this part, we examine the robustness of the NEAPT algorithm in dynamic scenarios. The total execution time was 500 BIs. We assumed that 10 sensor nodes were always active during the 500 BIs. Fifteen more sensor nodes became active at the 101st BI, and returned to being inactive at the 401st BI. Another 20 sensor nodes became active at the 201st BI, and returned to being inactive 100 BIs later. Hence, there were 45 sensor nodes in total. Since all the considered parameter tuning strategies were based on previous communication outcomes, either inaccurate reliability estimation or suddenly changed network conditions would cause opposite or overdue parameter tuning, which would lead to the fluctuation of the packet delivery ratio.

**Figure 5:**
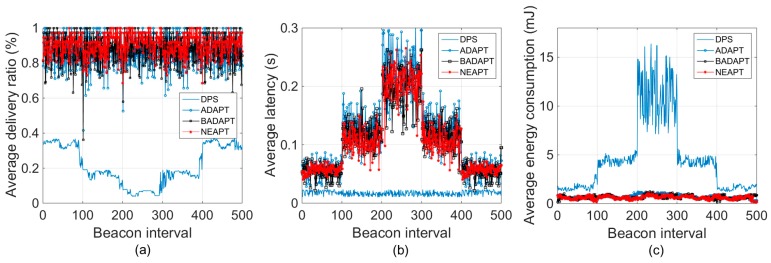
Delivery ratio, latency and energy consumption under dynamic conditions. (**a**) Delivery ratio. (**b**) Average latency. (**c**) Energy consumption.

The results presented in Figure 5 refer to one of ten sensor nodes that were always active. Figure 5a shows that the reliability of the DPS was always below 40%, whereas the delivery ratios of ADAPT, BADAPT, and NEAPT were above 80% most of the time. Throughout the entire 500 BI simulation, the ratio of failing to meet the reliability requirement with NEAPT was 5.6%. For ADAPT and BADAPT, their ratios were 21.2% and 17.4%, respectively. It should be noted that the packet delivery ratio of ADAPT and BADAPT decreased sharply at the 101st and the 201st BI due to the sudden change of network condition. In detail, for ADAPT, we observe that the packet delivery ratios at two sudden changes were 42% and 53%. And the recovery time after each sudden change was around 7 BIs. As for BADAPT, we find the packet delivery ratios of two sudden changes were 36% and 58% and the recovery time was also around 7 BIs. While for NEAPT, no such serious packet delivery ratio decrease occurred throughout the entire simulation. The recovery time after each sudden change was around 5 BIs, which is lower than the recovery time of ADAPT and BADAPT. This is mainly due to the conservative parameter tuning method of NEAPT. These results show that NEAPT has better robustness than ADAPT and BADAPT under dynamic conditions. From Figure 5b,c, it is shown that ADAPT and BADAPT experienced almost the same energy consumption and latency performance under dynamic conditions. What’s more, it is also shown that NEAPT was able to provide a steadier performance under dynamic conditions, in terms of transmission latency and energy consumption, than ADAPT and BADAPT. This result not only proves the ability of NEAPT in providing qualified and steady performance in dynamic conditions, but also presents its advantage of conservative parameter tuning in buffering sudden changes of network condition.

### 5.3. Applicable Network Scales of NEAPT

A series of experiments were performed to specify the application range of the proposed NEAPT on a network scale. Within these experiments, the required packet delivery ratio $R_{req}$ was fixed at 80%. The time interval of each sensor node’s successive data packet ranged from 3 s to 7 s. The scale of the network ranged from 5 nodes to 95 nodes. Simulations with different network scales were separately executed over 100 BIs. In these simulation experiments, the sensor nodes first adopted ADAPT as their parameter tuning algorithm. After each experiment, values of *macMaxCSMABackoffs* that satisfied the reliability requirement were collected. The average value of each experiment’s collected values were calculated and recorded. Then, under the different network scales, the suggested values of *macMaxCSMABackoffs* by NEAPT were analytically obtained and recorded.

**Figure 6:**
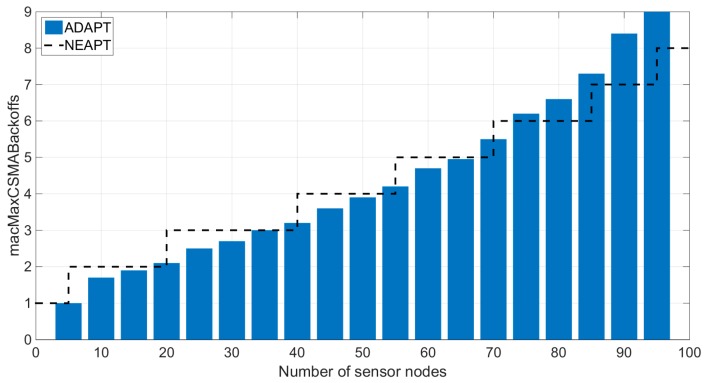
Optimal values of *macMaxCSMABackoffs* derived from the analytical model and simulation.

Figure 6 indicates that the NEAPT recommended value behaved as an upper bound of the ADAPT simulated result when the network scale was less than 70 sensor nodes. As the number of sensor nodes exceed 70, the NEAPT recommended value could no longer be taken as the upper bound of the simulated value. This could be explained by the feasibility of the proposed reliability analysis. As the number of sensor nodes grows, the arrival of data packets cannot be approximated as a Poisson process any more. Hence, the proposed Poisson based reliability analysis is not appropriate for WSNs with more than 70 sensor nodes. Based on these experiments, we found that NEAPT is feasible for small and medium WSNs.

## 6. Conclusions

To provide adequate and steady performances for WSNs in both stationary conditions and dynamic conditions, this paper proposes a heuristic algorithm called NEAPT. It is a fully distributed algorithm and can work without any predefined information or acknowledgement. Unlike directly taking reliability as the index of the network condition, NEAPT proposes another evaluation index of the network condition for sensor nodes, that is, the *equivalent node number*. NEAPT can reliably recognize significant violations from general tiny fluctuations of reliability, by taking both reliability and the *equivalent node number* into consideration. With NEAPT, the sensor nodes can evaluate the surrounding network’s condition and perform the parameter tuning process independently. Simulation results demonstrated that NEAPT could provide an adequate and steady performance in terms of reliability, time efficiency, and energy efficiency, as well as improve the robustness of network for dynamic conditions. Finally, NEAPT is proven to be suitable for the small and medium scale WSNs.

For future work, we should gain deep insights into several problems, such as: (a) the existence of hidden nodes should be considered and coping mechanisms for it should be added, and (b) the reliability analysis of large scale WSNs and dense WSNs should be considered to expand the feasibility of proposed parameter tuning algorithm. Also, we intend to implement the proposed parameter tuning algorithm using a real hardware testbed and to add new mechanisms for mobile WSNs scenarios.

## Figures and Tables

**Table 1:** Carrier sense multiple access with collision avoidance (CSMA/CA) parameters and values [27].

Parameter	Values		Description
	Default	Range	
CW_0_	2		The initial length of contention window
*macMaxCSMABackoffs*	4	0–5	Maximum number of backoff stages-1
*macMaxBE*	5	3–8	Maximum backoff window exponent
*macMinBE*	3	0–7	Minimum backoff window exponent
*aUnitBackoffPeriod*	320 μs		Time duration of unit backoff slot

**Table 2:** Parameters for simulation. BO—beacon order; SO—superframe order; RX—Receiving; TX—Transmitting.

Parameter	Value
Bit rate	250 kbps
Packet size	120 bytes
BO, SO	13, 10
Target reliability ($p_{req}^{S}$)	80%
The threshold of network change ($\delta_{N}$)	2
*macMinBE^min^, macMinBE^max^*	1, 7
*macMaxBE^max^*	10
*macMaxCSMABackoffs^min^, macMaxCSMABackoffs^max^*	1,10
Power consumption in RX, TX, idle, sleep	56.4, 52.2, 1.28, 0.06 mW

## Appendix A

In this appendix we prove that the aggregate packet arrival rate of one channel can be approximated as the Poisson distribution, which is introduced in Section 5.

We assume that each sensor device has a fixed but different data packet arriving interval. The data packet arriving interval of the sensor node *i* is *T_i_* seconds, which consist of $n_{T}^{i}$ sequential time slots. For sensor node *i*, the probability that there comes a data packet at every time slot is ${\left(n_{T}^{i}\right)}^{-1}$. For one channel shared by $N^{C}$ devices, let’s define the LCM (lowest common multiple) of all of the devices’ data packet arriving interval as the period of this channel $n_{T}^{C}$. At each time slot, the probability that there comes a data packet needed to be transmitted on this channel is as follows:

(A1) $$p^{C}=1-\prod_{i=1}^{N^{C}}\left(1-(n_{T}^{C}/n_{T}^{i})/n_{T}^{C}\right).$$

For each sensor node, its successive data packets usually have a very strong correlation, since each of the nodes usually report to the PAN coordinator periodically. However, for one channel shared by several sensor nodes, the correlation between its two successive data packets is much weaker. Since successive data packets that are arriving at one channel always come from different sensor nodes, they may be completely irrelevant regardless of the terms of external the surroundings or application objects. Hence, it is more adequate to approximate the case that the k data packets arrive at one channel within the $n_{T}^{C}$ time slots as a binomial process. The corresponding probability is as follows:

(A2) $$p_{k,n_{T}^{C}}^{C}=\left(\frac{k}{n_{T}^{C}}\right){\left(p^{C}\right)}^{k}{\left(1-p^{C}\right)}^{n_{T}^{C}-k}.$$

According to (A1), for a given channel period, as the duration of each time slot decreases, the value of $n_{T}^{C}$ increases, while the value of $p^{C}$ decreases. Hence, we can get the limit of $p_{k,n_{T}^{C}}^{C}$,

(A3) $$\underset{n_{T}^{C}\to \infty ,p^{C}\to 0}{\lim}p_{k,n_{T}^{C}}^{C}=\underset{n_{T}^{C}\to \infty ,p^{C}\to 0}{\lim}\frac{{\left(n_{T}^{C}p^{C}\right)}^{k}}{k!}{\left(1-p^{C}\right)}^{-\frac{\left(n_{T}^{C}p^{C}\right)}{p^{C}}}\frac{1}{{\left(1-p^{C}\right)}^{k}}.$$

As $n_{T}^{C}$ increases and $p^{C}$ approximates to 0, ${\left(1-p^{C}\right)}^{-\frac{1}{p^{C}}}$ approximates to e and ${\left(1-p^{C}\right)}^{-k}$ approximates to 0. The limit of $p_{k,n_{T}^{C}}^{C}$ can be furtherly expressed as follows:

(A4) $$\underset{n_{T}^{C}\to \infty ,p^{C}\to 0}{\lim}p_{k,n_{T}^{C}}^{C}=\underset{n_{T}^{C}\to \infty ,p^{C}\to 0}{\lim}\frac{{\left(n_{T}^{C}p^{C}\right)}^{k}}{k!}e^{-(n_{T}^{C}p^{C})}.$$

Let the product of $n_{T}^{C}$ and $p^{C}$ be λ, as follows:

(A5) $$\lambda =n_{T}^{C}\cdot (1-\prod_{i=1}^{N^{C}}\left(1-(n_{T}^{C}/n_{T}^{i})/n_{T}^{C}\right)),$$

according to (A4), we can get the distribution of λ, which approximates to the Poisson distribution, as follows:

(A6) $$\underset{n_{T}\to \infty ,p_{c}\to 0}{\lim}p_{C}^{k,n_{T}}=\underset{n_{T}\to \infty ,p_{c}\to 0}{\lim}\frac{{\left(\lambda \right)}^{k}}{k!}e^{-(\lambda )}.$$

In the slotted CSMA/CA algorithm, the duration of the unit time slot is 320 μs. For each of the considered sensor nodes, the time interval between its two successive data packets is counted in seconds. The value of the channel period $n_{T}^{C}$ is at the level of hundreds to thousands, accordingly. Hence, the aggregate packet arrival rate of one channel is reasonable to be approximated as a Poisson process.
